# TockyLocus: quantitative analysis of flow cytometric fluorescent timer data in Nr4a3-Tocky and Foxp3-Tocky mice

**DOI:** 10.1093/biomethods/bpaf060

**Published:** 2025-08-26

**Authors:** Masahiro Ono

**Affiliations:** Department of Life Sciences, Imperial College London, Imperial College Road, London SW7 2AZ, United Kingdom

**Keywords:** Fluorescent Timer, Tocky, Foxp3, Nr4a3, T cell receptor signalling, flow cytometry

## Abstract

Fluorescent Timer proteins undergo a time-dependent shift from blue to red fluorescence after translation, providing a temporal record of transcriptional activity in Timer reporter systems. While Timer proteins are well suited for studying dynamic cellular processes such as T cell activation using the Timer-of-Cell-Kinetics-and-Activity (Tocky) framework, quantitative analysis of Timer-based flow cytometry data has yet to be fully standardized. In this study, we optimize quantitative analysis methods for the key parameter within the Tocky framework, Timer Angle, and introduce TockyLocus, an open-source R package that implements a five-category scheme based on biologically grounded angular intervals (designated as Tocky Loci). This approach is validated using both simulated and experimental datasets and enables downstream statistical testing and visualization of transcriptional dynamics in flow cytometry data. Using computational modelling of Timer protein kinetics, we define transcriptional dynamics in relation to key anchoring points in Timer Angle values at $0^{{}^{\circ}}$, $45^{{}^{\circ}}$, and $90^{{}^{\circ}}$. Comprehensive simulations with synthetic spike-in datasets further demonstrate the robustness of the five-locus approach, which captures the three key points and the intermediate regions between these points. Building on the TockyPrep preprocessing framework, we systematically evaluated categorization schemes ranging from three to seven loci on real-world datasets from Nr4a3-Tocky and Foxp3-Tocky mice. The five-locus model emerged as optimal, showing significant advantages in balancing biological interpretability and statistical robustness. Optimized algorithms implemented in the TockyLocus package now standardize quantitative analysis of Timer Angle data, enabling reproducible interpretation without reliance on arbitrary gating or complex assumptions. In summary, the five-locus categorization of Timer Angle data effectively links underlying biological dynamics to the percentage of cells in each Tocky Locus, providing a robust and interpretable framework for investigating transcriptional dynamics in immunology and related fields.

## Introduction

Fluorescent Timer proteins are a class of genetically encoded reporters that undergo a spontaneous spectral shift from blue or green to red emission over time. These proteins have been used to analyse processes such as gene activation, differentiation, and cellular responses to signalling. Unlike conventional fluorescent proteins with long half-lives (e.g. GFP), FT proteins such as Fast-FT, Medium-FT, and tandem-timer constructs exhibit maturation kinetics on the order of hours, enabling the resolution of recent transcriptional activity [1, 2]. Notably, Fast-FT is compatible with flow cytometric analysis, shifting its emission spectrum from CFP-like blue fluorescence (Timer Blue) to mCherry-style red fluorescence (Timer Red) in 4 h as its maturation half-life, and thus enabling the analysis of rapid changes in cellular and molecular dynamics [1, 3].

Previously, the Ono group reported the Timer-of-Cell-Kinetics-and-Activity (Tocky) framework, which analyses Timer fluorescence in immunological and cell biological contexts to understand temporal changes in cellular activities using Fluorescent Timer reporter mouse strains, with Fast-FT as the reporter gene [3, 4]. The Nr4a3 Fluorescent Timer reporter, or Nr4a3-Tocky mice, analyse T cell activities by monitoring the temporal activity of the TCR signal downstream gene Nr4a3, enabling *in vivo* tracking of T cell dynamics following T cell receptor (TCR) signalling [3, 5–8]. In addition, Foxp3-Tocky was developed to analyse the transcriptional dynamics of Foxp3, a key T cell regulatory gene [3, 9]. Although formerly categorized as a regulatory T-cell-specific gene, recent work has elucidated the dynamic regulation of Foxp3 expression across a wide range of CD4 T cells [10–12].

To systematically understand Timer maturation dynamics, we previously proposed a data transformation method using a trigonometric transformation to derive the Timer Angle, where fluorescence signals are mapped onto a polar coordinate system such that the Timer Blue axis corresponds to 0°, and the Timer Red axis corresponds to 90° [3].

The Timer Angle approach has provided opportunities to identify unique phases of TCR signal dynamics using Nr4a3-Tocky: (i) T cells that have recently received TCR signalling are prevalent at Timer Angle 0°; (ii) T cells recognizing antigen and receiving TCR signalling approach Timer Angle 45°; and (iii) T cells that have recognized antigen and received TCR signalling and have been removed from antigen approach 90° [3].

In addition, the Timer Angle approach has enabled the identification of unique Foxp3 transcription dynamics using Foxp3-Tocky: (a) T cells that have newly activated Foxp3 transcription are prevalent at Timer Angle 0°; (b) T cells that have persistently transcribed Foxp3 exhibit approximately Timer Angle 45°; and (c) T cells that have once activated Foxp3 transcription and then terminated it show Timer Angle 90° [3, 9].

Thus, the Timer Angle approach has opened a new avenue for coherently understanding Timer fluorescence data. However, Timer Angle data present new challenges. First, it spans a continuous range from 0° to 90° and is highly dynamic, and may be significantly skewed towards either 0° or 90°, still posing significant challenges for statistical and quantitative analysis. Second, the nature of this angular distribution, which may be unimodal, bimodal, or diffuse, renders manual gating both impractical and potentially misleading. In addition, a robust statistical analysis of Timer Angle data has not been formally established. Third, it remains difficult to relate continuous Timer Angle values directly to biological interpretation, particularly given the unique and often skewed distributions observed.

To address these challenges, previous work has established methods for data transformation and normalization (TockyPrep) [13], dimensionality reduction and trajectory inference (TockyDevelopment) [14], and machine learning approaches using Convolutional neural network analysis and Random Forest [15]. However, a key analytical bottleneck remains unresolved: the need for the most optimized and fundamental approach to interpret continuous Timer Angle data without relying on data-oriented clustering or complex machine learning models. Especially, it is urgently required to establish a simple yet robust method to associate Timer Angle with biologically meaningful categories.

Discretization of continuous fluorescence measurements is a fundamental analytical step in flow cytometry [16]. In typical flow cytometric analyses, data categorization through gating is used to derive proportional values representing the prevalence of defined cell populations and is essential for comparing group-specific features and applying statistical hypothesis testing [17]. However, the currently prevalent method for categorization, manual gating, involves the subjective and often arbitrary delineation of populations via graphical interfaces. This approach is increasingly impractical and unreliable in the context of modern flow cytometry, where datasets are becoming increasingly complex and multidimensional [18, 19], and even 2D Timer fluorescence data suffer significantly from the arbitrariness and biases inherent to manual gating [15].

Although considerable work has addressed the challenges of high-dimensional data and the development of automated clustering algorithms, another important issue remains less recognized: how to standardize categorization when distributions are unimodal, diffuse, or severely skewed. Such distributions are common, and Timer Angle data are a clear example, and are difficult to handle consistently with either manual or automated gating. Computational clustering introduces stochastic elements [20–22] and unnecessarily complicates the analysis of fundamentally one-dimensional data. Moreover, this problem is not resolved by batch correction or sample-alignment techniques such as landmark registration or quantile normalization [23, 24], which address between-sample variability but do not define biologically meaningful categories. In the context of Fluorescent Timer data, we previously proposed a five-locus classification scheme based on angular partitioning [3]. However, the assumptions and boundaries of that scheme were empirically determined and had not been formally evaluated until the present study.

In this study, we present TockyLocus, a computational tool that implements a systematic classification scheme for discretizing FT data into angular loci. We evaluate the performance of multiple categorization schemes using both synthetic and experimental datasets and demonstrate that the five-locus model offers an optimal balance between resolution and interpretability. Furthermore, the tool includes integrated modules for data visualization and statistical testing, enabling hypothesis-driven analysis of temporal dynamics in FT-labelled cell populations. Together with TockyPrep, the TockyLocus package provides a complete and reproducible pipeline for the analysis of FT data in flow cytometry.

We used datasets from Nr4a3-Tocky and Foxp3-Tocky, which are ideal systems to showcase the TockyLocus method because their dynamic transcriptional activities generate unique and nuanced Timer Angle distributions associated with specific Timer Angle values, as described above. This challenges conventional statistical analyses, making them biologically and technically rigorous test cases for developing a data categorization approach for Timer Angle, or the Tocky Locus approach.

## Materials and methods

### Implementation of Tocky locus analysis

To apply Tocky Locus analysis to flow cytometric data, the following analysis pipeline is used:

#### Data preprocessing of Timer fluorescence data

This part uses TockyPrep [25] and involves the following steps:

- **Timer Thresholding:** Establishes Timer fluorescence intensity thresholds to define Timer-positive cells, excluding autofluorescence.
- **Timer Normalization:** Adjusts Timer Blue and Timer Red fluorescence intensities to correct for systematic differences between channels and across samples, ensuring comparability.
- **Trigonometric Transformation:** Converts normalized Timer Blue and Red fluorescence values into polar coordinates, yielding the following two derived parameters:
  – **Timer Angle (**θ**)** represents the angular orientation in the Blue–Red fluorescence space, reflecting the relative maturation state of the Timer protein.
  – **Timer Intensity (*d*)** represents the radial magnitude of the fluorescence vector, capturing the overall level of Timer protein expression.

#### Tocky Locus: data categorization of Timer Angle data

The data categorization method addressed in this study partitions Timer Angle data into distinct categories, known as Tocky Loci:

-
$\theta =0^{{}^{\circ}}$ **(New):** Corresponds to cells with Timer Blue+Red–, indicating newly initiated transcription with immature Timer protein.
-
$\theta =90^{{}^{\circ}}$ **(Arrested):** Corresponds to cells with Timer Blue^–^Red^+^, reflecting cells that have ceased Timer transcription and contain only mature Timer protein.

The intermediate regions between New and Arrested are partitioned as follows in the standard five-locus scheme:

-
$0^{{}^{\circ}}<\theta \leq 30^{{}^{\circ}}$ **(NP-t):** New-to-Persistent transitioning, indicating recent initiation of transcription progressing towards sustained activity.
-
$30^{{}^{\circ}}<\theta \leq 60^{{}^{\circ}}$ **(Persistent):** Represents sustained transcription with balanced levels of Timer Blue and Red fluorescence, typically centred around $45^{{}^{\circ}}$.
-
$60^{{}^{\circ}}<\theta <90^{{}^{\circ}}$ **(PA-t):** Persistent-to-Arrested transitioning, indicating declining or recently terminated transcription with increasing Timer Red predominance.

For other numbers of loci (e.g. 3, 4, 6, or 7), the intermediate angle space is divided into equal-width segments between the anchor points at 0° and 90°.

#### Methods for percentage of cells per locus

Two methods for calculating the distribution of cells within each Tocky Locus are implemented. The default method, the Percentage-Parent, calculates the percentage of cells within each locus relative to the total cell population analysed. This metric assesses the distribution and predominance of cells across the Tocky Loci within the population of interest. In contrast, the Percentage-Timer calculates the percentage of cells within the population of Timer-positive cells, thereby focusing on proportion analysis. This approach emphasizes the prominent Tocky Loci within Timer+ cells.

Percentage-Parent refers to the proportion of cells in each Tocky locus relative to a broader parent population of interest (e.g. total CD4 T cells). This metric is typically the primary choice in most *in vivo* analyses, where it provides context for how specific transcriptional states contribute to overall cellular composition.

In contrast, Percentage-Timer denotes the proportion of cells in each Tocky locus relative only to the Timer-positive cell population. This metric is useful for examining the relative distribution of Timer Angle states, highlighting subtle shifts in Timer dynamics independent of changes in total cell numbers.

*In vitro* T cell stimulation experiments usually involve purified T cell samples, meaning that Percentage-Parent and Percentage-Timer often yield similar results in those contexts. However, in complex tissues *in vivo*, the two measures can diverge significantly, and the choice between them depends on the specific biological question being addressed.

#### Visualization of Tocky Locus data

The use of line graphs with individual data points is recommended and implemented to effectively capture nuanced dynamics. This approach allows for the application of statistical methods that are both analytically rigorous and visually interpretable.

#### Statistical application to Tocky Locus data

Appropriate statistical analyses are applied to Tocky Locus categorized data. Currently, the Mann-Whitney test with *P*-value adjustment is implemented to ensure robust comparison across categories.

### Data preprocessing implemented in TockyPrep

Data preprocessing of Timer fluorescence data was performed using the TockyPrep package [25]. This analysis stage comprises two major processes:

1. Timer blue and red fluorescence signals are normalized based on statistics derived from gated negative control cells. Thresholds for red ($x_{\text{lim}.\text{red}}$) and blue ($y_{\text{lim}.\text{blue}}$) fluorescence are established either interactively or automatically using quantile-based methods. For each fluorescence channel, the Median Absolute Deviation (MAD) from the log-transformed fluorescence intensities of the gated negative control cells is computed. The normalized blue fluorescence ($B_{\text{norm}}$) and red fluorescence ($R_{\text{norm}}$) for each cell are calculated as follows:
  (1) $$B_{\text{norm}}=\frac{B_{\;\text{log}\;}-\max(B_{\;\text{log}\;,\;\text{neg}})}{\text{MAD}(B_{\;\text{log}\;,\;\text{neg}})},$$
   
  (2) $$R_{\text{norm}}=\frac{R_{\;\text{log}\;}-\max(R_{\;\text{log}\;,\;\text{neg}})}{\text{MAD}(R_{\;\text{log}\;,\;\text{neg}})},$$
  where $B_{\;\text{log}\;}$ and $R_{\;\text{log}\;}$ are the log-transformed blue and red fluorescence intensities of individual cells, and the subscript ‘neg’ refers to the negative control cells.
2. To capture the temporal progression of the Timer fluorescence maturation from blue to red, the normalized fluorescence data are transformed into polar coordinates, yielding two new parameters: Timer Intensity (*I*) and Timer Angle (θ) [3]. The Timer Intensity *I* represents the overall expression level of the Timer protein and is calculated as: $I=\sqrt{B_{\text{norm}}^{2}+R_{\text{norm}}^{2}}$. The Timer Angle θ reflects the maturation state of the Timer protein, corresponding to the time elapsed since expression. It is computed using the arccosine function:
  (3) $$\theta =\arccos\left(\frac{B_{\text{norm}}}{I}\right)\times \left(\frac{180}{\pi }\right),$$
  Timer Angles range from $0^{{}^{\circ}}$ (indicative of pure blue fluorescence) to $90^{{}^{\circ}}$ (indicative of pure red fluorescence).
3. Timer Angle data are further analysed by functions implemented in the TockyLocus package.

### Datasets

#### Nr4a3 Tocky dataset

The dataset was derived from flow cytometric analysis of *in vitro* cultured T cells from Nr4a3-Tocky::OT-II double transgenic mice [3]. The OT-II transgenic T-cell receptor recognizes the Ova_(323-339)_ peptide. Upon TCR signalling, Nr4a3-Tocky T cells express the Fluorescent Timer. Briefly, OT-II Nr4a3-Tocky T cells were activated using Ova (1 μM) in the presence of antigen-presenting cells. Time-course analysis was performed. The Timer fluorescence data from this dataset were partially included in the TockyPrep package. Table 1 shows the group identities of samples within the Nr4a3 Tocky dataset.

**Table 1.** Summary of groups, timer points, and treatments.

Group	Time (h)	Treatment
0	0	Stimulation from 0 h
4	4	Stimulation from 0 h
8	8	Stimulation from 0 h
12	12	Stimulation from 0 h
16	16	Stimulation from 0 h
24	24	Stimulation from 0 h
32	32	Stimulation from 0 h
48	48	Stimulation from 0 h
32.a	32	Stim. till 24 h, then suspended
48.a	48	Stim. till 24 h, then suspended

#### Anti-OX40 dataset

This dataset originated from flow cytometric analysis of ear skin-infiltrating CD4^+^ T cells from Foxp3-Tocky mice subjected to allergic skin inflammation and treated with immunotherapy [9]. Briefly, the oxazolone-induced contact hypersensitivity (CHS) model was applied to Foxp3-Tocky mice aged five to ten weeks. Mice were divided into a control group (receiving isotype rat IgG1 [MAC221]) and an anti-OX40 antibody group (receiving 0.5 mg anti-OX40 [OX86] antibody), with four mice in the control group and three in the anti-OX40 group. Mice were sensitized with oxazolone on the abdominal skin on day –5, challenged on the ears on day 0, and analysed on day 5. Immunotherapy was administered on days 0 and 3 via intraperitoneal injection.

#### CNS2 KO Foxp3-Tocky dataset

This dataset originated from flow cytometric analysis of lymph node CD4^+^ T cells from Foxp3-Tocky mice, either wild-type or carrying a CRISPR-mediated knockout of the CNS2 enhancer within the Foxp3 Timer transgene [15]. Briefly, embryos derived from Foxp3-Tocky mice were subjected to pronuclear injection for CRISPR editing. A PCR- and sequence-confirmed CRISPR subline was established. Superficial lymph node cells were obtained from littermates of Foxp3-Tocky (wild-type) and CNS2 KO Foxp3-Tocky parents and analysed using a BD FACSymphony A3 flow cytometer. The dataset used for this analysis was gated on CD4^+^ T cells.

#### Computational simulation for protein maturation kinetics

Computational simulations were performed using the R package deSolve (version 1.40) [26] to model the biochemical kinetics of fluorescent Timer proteins under two transcriptional scenarios: a constant transcription model and a transient transcription model. These simulations recapitulate how transcriptional activity translates into the maturation dynamics of Timer Blue and Timer Red fluorescence.

##### Reaction model

The underlying biochemical pathway involves the following reactions, parameterized by experimentally derived rate constants [1, 3, 9] (per hour):

- Transcriptional activity (*X*) produces intermediate compound *C* at rate d=0.3.
- *C* converts to immature Timer Blue (*B*) at rate $k_{b}=8.7$.
- *B* matures to intermediate form *I* at rate $k_{i}=0.78$.
- *I* converts to mature Timer Red (*R*) at rate $k_{r}=0.14$.
- *R* degrades at rate s=0.048.

##### Constant transcription model

This model assumes transcription (*X*) is constant over time, simulating steady-state transcriptional activity. Simulations were run over time intervals from 0 to 168 h, for initial transcription levels *X* varying between 100 and 1000 in increments of 20. Outputs included the concentrations of Timer Blue (*B*) and Timer Red (*R*), which were further transformed by log-scale for visualization.

##### Transient transcription model

To represent transient gene activation (e.g. as observed for *Nr4a3* [3]), a windowed Gaussian spike function was implemented for *X*:


$$X(t)=\{\begin{array}{cc} c_{\text{spike}}\cdot \;\text{exp}(-k_{\text{decay}}\cdot {\left(t-t_{\text{peak}}\right)}^{2}), & t_{\text{on}}\leq t\leq t_{\text{off}}\\ 0, & \text{otherwise} \end{array}$$

with parameters:

-
$c_{\text{spike}}=10$

-
$t_{\text{peak}}=2.5$ h
-
$t_{\text{on}}=0.5$ h, $t_{\text{off}}=6$ h
-
$k_{\text{decay}}=1.8$

-
$k_{\text{deg}}=0.28$ (degradation of *X*)

Simulations were run from 0 to 48 h at 0.1-h intervals. To introduce biological variability, synthetic populations were generated by randomly scaling *B* and *R* fluorescence intensities between 10-fold and 100-fold.

##### Post-processing and transformation

Simulated Timer Blue (*B*) and Red (*R*) fluorescence values were:

- Log-transformed using ${\;\text{log}\;}_{10}(x+1)$.
- Normalized across channels to correct systematic biases.
- Converted to Timer Angle (θ) and Timer Intensity (*d*) via polar coordinate transformation:
  $$\theta =\arccos\left(\frac{B}{\sqrt{B^{2}+R^{2}}}\right)\cdot \frac{180}{\pi }$$

##### Visualization

The simulations produced:

- Contour plots of Timer Blue vs. Timer Red fluorescence over various time intervals.
- Ridge plots and violin plots displaying distributions of Timer Angle across time.
- Overlay comparisons between experimental and simulated Timer Angle trajectories for validation.

This simulation framework enabled direct testing of how transcriptional kinetics generate distinct Timer Angle distributions, informing the design and interpretation of the Tocky Locus categorization approach.

##### Software

The TockyLocus package imports functions from the R packages ggplot2 [27] and ggridges [28] for graph production, the package RColorBrewer [29] for generating colours, and TockyPrep [25] for data preprocessing of Timer fluorescence data. The Shapiro-Wilk normality test was performed using the package stats [30]. All analysis was performed using R (version 4.4.1) [30].

## Implementation

### Overview

**Figure 1:**
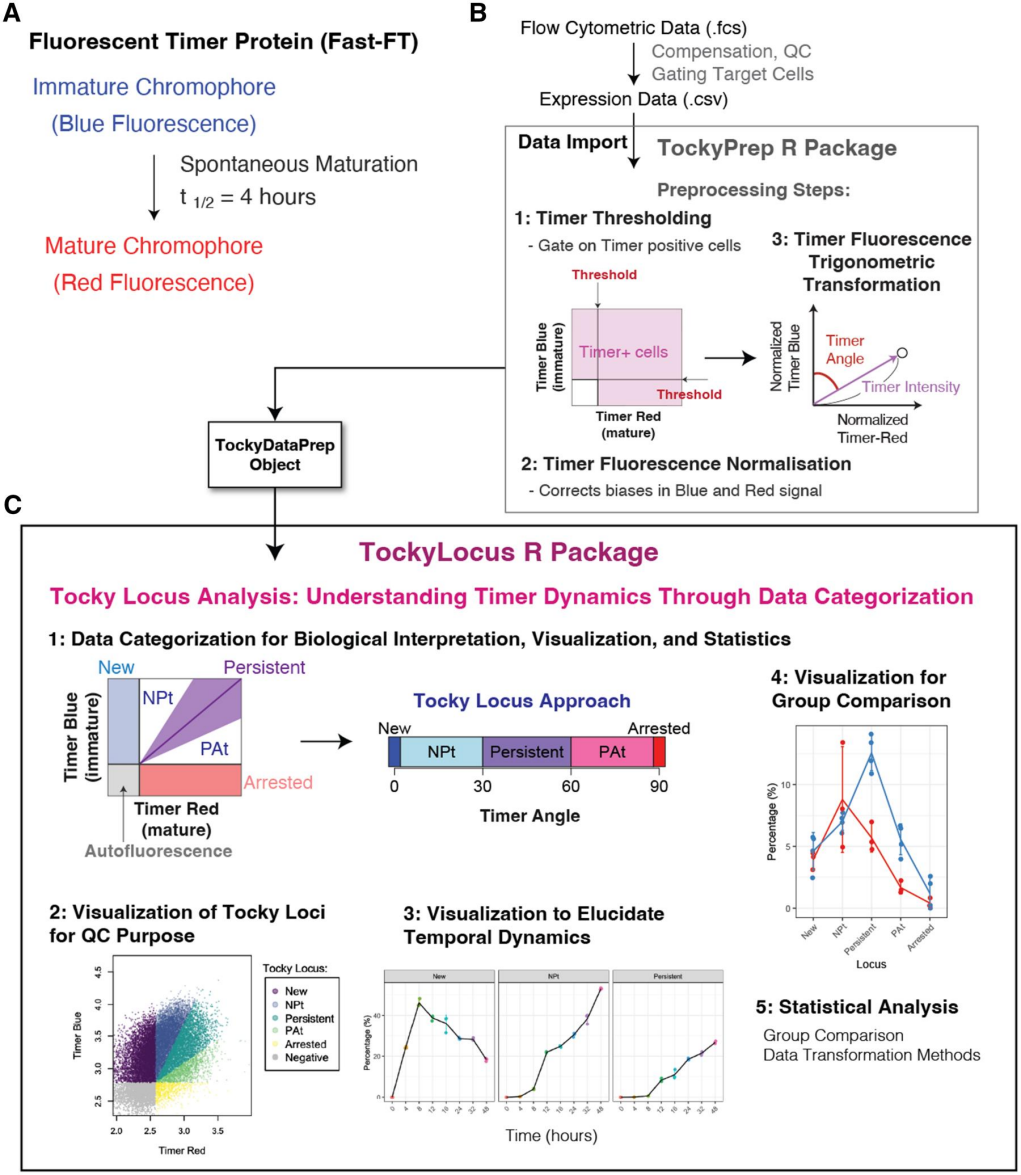
Overview of TockyLocus Implementation and its relationship with the TockyPrep package. (**A**) The maturation dynamics of Fluorescent Timer protein (Fast-FT). (**B**) The data import and preprocessing using the TockyPrep package, which applies Timer thresholding, Timer fluorescence normalization, and Timer fluorescence trigonometric transformation. The schematic figure was adapted from our previous publication [13]. (**C**) The preprocessed data is stored as TockyDataPrep S4 object, which is further analysed using the TockyLocus package, which provides five major functions as depicted. The schematic figures for the five loci were adapted and modified from our previous publication [3].

This section provides the overview for the implementation of Tocky Locus analysis algorithms in the TockyLocus R package (Fig. 1).

To unravel the dynamics of Fluorescent Timer protein (Fig. 1A), the TockyLocus package is implemented with unique functions and analyses preprocessed data using the TockyPrep package. The TockyPrep package, which applies Timer thresholding, Timer fluorescence normalization, and Timer fluorescence trigonometric transformation (Fig. 1B). Note that the preprocessed data is stored as TockyDataPrep S4 object, which is further analysed using the TockyLocus package.

The TockyLocus package provides five major functions:

1. Data categorization using the five Tocky loci.
2. Visualization of Tocky Loci in flow cytometric plots for quality control (QC) purposes.
3. Visualization of temporal dynamics using locus-wise plots.
4. Visualization for group comparisons.
5. Statistical analysis methods.

### Statistical testing methods

The TockyLocus package implements three statistical methods to compare Tocky locus percentages between experimental groups.

First, a non-parametric Wilcoxon rank-sum test (Mann-Whitney U test) is available for locus-wise comparisons without assuming normality of the data. This test assesses whether the distributions of percentage values differ between two groups, and is applied individually to each locus. Resulting *P*-values are adjusted for multiple testing, typically using the Benjamini-Hochberg (BH) method, with significance determined at an adjusted threshold of 0.05.

Alternatively, parametric testing is supported through the arcsine square root (ASR) transformation, which stabilizes variances and helps approximate normality when analysing proportion data bounded between 0 and 100% [31, 32]. The transformation is defined as:


$$\text{Transformed}\_\text{Percent}=\arcsin\left(\sqrt{\frac{\text{Percent}}{100}}\right).$$

Following the transformation, the Shapiro-Wilk test is performed to evaluate the normality of the transformed values. If normality is not rejected (*P*-value > .05), independent two-sample t-tests are conducted for each locus to compare group means. *P*-values from these tests are corrected for multiple comparisons, and loci are considered significant at an adjusted *P*-value below .05.

As an alternative to the ASR method, the package also offers the logit transformation for percentage data [31, 33]. This approach is particularly suitable for avoiding distortion near the bounds of 0% and 100%. The transformation is defined as:


$$\text{Transformed}\_\text{Percent}=\text{log}\;\left(\frac{\text{Percent}+c}{100-\text{Percent}+c}\right),$$

where c is a small constant (e.g. 0.001) added to prevent undefined values. After applying the logit transformation, normality is assessed using the Shapiro-Wilk test. If normality is confirmed, *t*-tests are performed per locus, with *P*-values adjusted for multiple testing as described above.

These complementary statistical approaches ensure robust analysis of Tocky Locus data under various distributional assumptions, allowing both non-parametric and parametric options depending on the data characteristics.

#### Multiple testing correction

For all statistical tests, *P*-values are adjusted to account for multiple comparisons across the five loci. The default method used is the Benjamini-Hochberg procedure, which controls the false discovery rate (FDR). Other adjustment methods available in the p.adjust function in R, such as Holm or Bonferroni corrections, can also be specified.

## Results

### Limitations of common mean fluorescence intensities-based methods

Using the Nr4a3 Tocky dataset [3], we aimed to refine methods for quantitatively analysing Timer Angle dynamics. Table 1 outlines the group identities and analysis time points. The Timer fluorescence data were converted into Timer Angle and Intensity using the TockyPrep package, which facilitated a detailed assessment of the dynamics involved.

**Figure 2:**
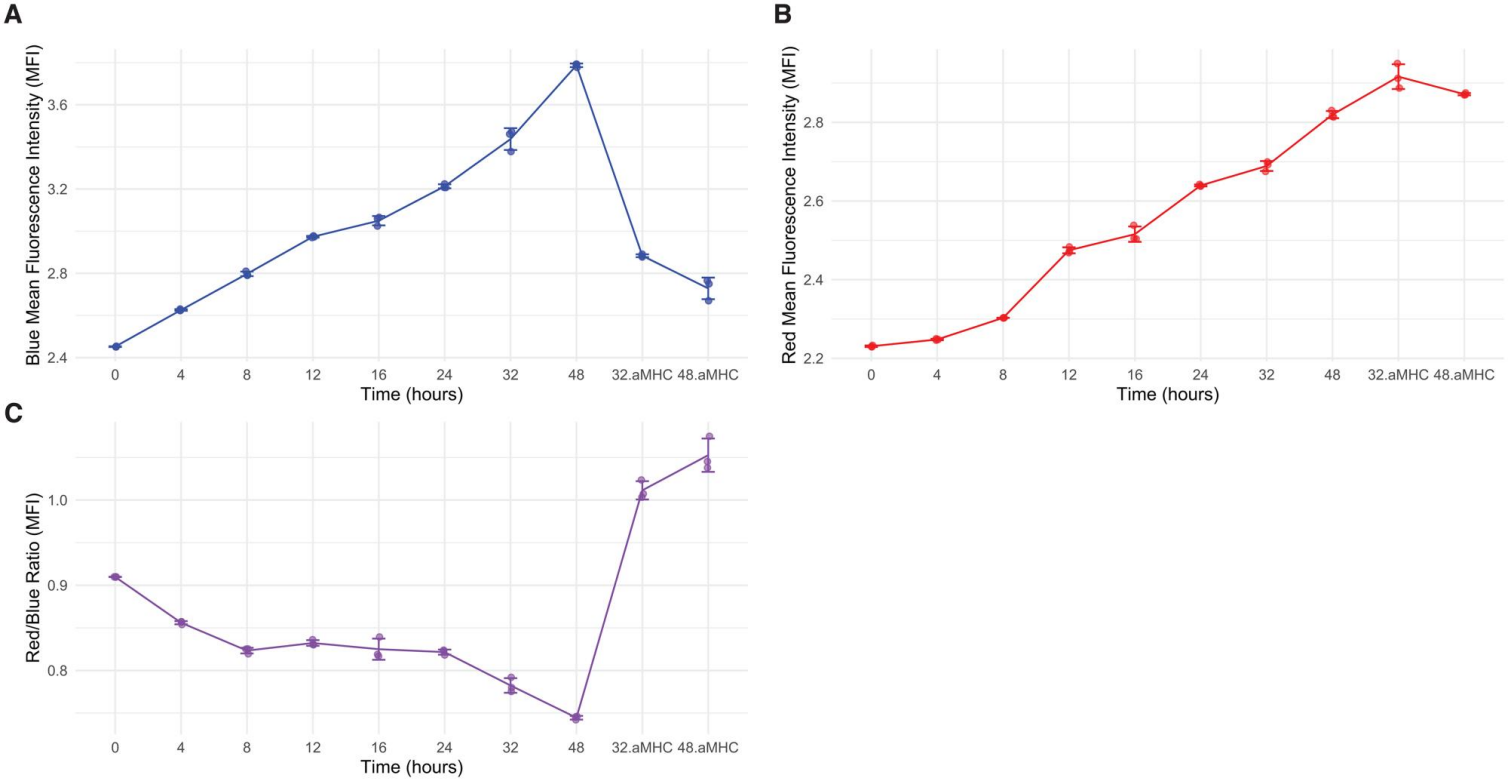
Limitations of Common Approach to Fluorescent Timer Data using Mean Fluorescence Intensities (MFI). Visualization of the Nr4a3 Tocky dataset showing (**A**) MFI of Timer-Blue, (**B**) MFI of Timer-Red, and (**C**) the ratio of Timer-Red to Timer-Blue MFI over time

Initially, we examined the traditional approach to Fluorescent Timer data, which relies on the mean fluorescence intensities (MFI) of Timer Blue and Red fluorescence [34–40]. This approach was applied to the Nr4a3 Tocky dataset (Fig. 2). We observed that the MFI for both Blue and Red fluorescence increased following antigen stimulation. However, while the removal of antigen stimulation resulted in a decrease in Blue MFI, the Red MFI remained unaffected. Consequently, the ratio of Blue to Red MFI steadily declined up to 48 h post-stimulation but surged upon cessation of stimulation.

This approach revealed significant limitations:

1. Comparing Timer Blue and Red fluorescence directly is problematic without adequate normalization and preprocessing, making it difficult to draw reliable conclusions from raw MFI data.
2. Consequently, interpretations based on the Red-to-Blue MFI ratio are inherently limited, as they fail to account for the underlying biological variances and the effects of antigen presence or absence.

### Strengths and limitations of timer angle approach using 2D and density plots

**Figure 3:**
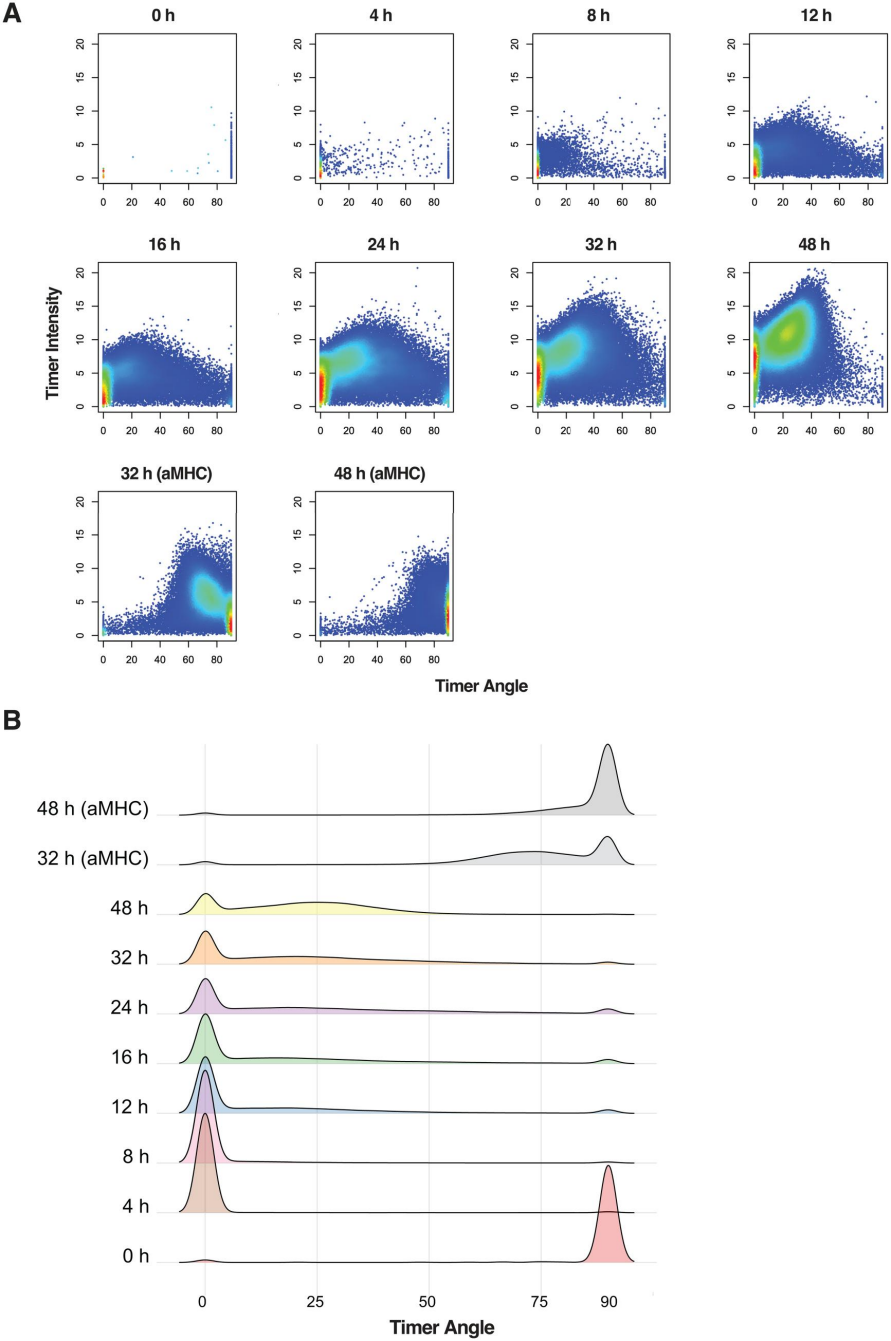
Timer Angle Approach to Analyse Dynamics of Activated T-cells. The TockyPrep package was utilized to normalize and transform Timer fluorescence data into Timer Angle and Timer Intensity. (**A**) The function plot_tocky from TockyPrep was employed to generate the Timer Angle vs Intensity plots. Refer to Table 1 for sample definitions. (**B**) Density plot of Timer Angle using the function plotAngleDensity. The plot visualizes the distribution of Timer Angles within the Nr4a3 dataset, highlighting major concentrations and transitions

We then applied the Tocky approach to analyse the Nr4a3 dataset. The data preprocessing was handled by the timer_transform function within the TockyPrep package, which includes steps such as Timer Thresholding, Timer Fluorescence Normalization, and Trigonometric Transformation. These steps effectively converted the Timer fluorescence data into quantifiable Timer Angle and Timer Intensity metrics, which are crucial for further analysis (Fig. 3A).

Despite these advancements, challenges remain in how to analyse the transformed data effectively, particularly in terms of interpreting and utilizing these metrics for biological insights.

Next, we assessed the effectiveness of density plots for visualizing and analysing the data. While density plots provide a visual representation of the dynamics of Timer Angle progression, they are limited in their ability to offer quantitative insights. Such visualizations illustrate trends and distributions but do not replace quantitative analysis methods that can offer more definitive conclusions (Fig. 3B).

Note that, although the time point 0 sample is dominated by Timer Angle $90^{{}^{\circ}}$ cells in the density plot (Fig. 3B), the number of these cells is indeed small (Fig. 3). This accumulation of small numbers of cells in the time point 0 sample is due to naturally occurring Timer+ cells in Nr4a3-Tocky mice including memory-phenotype T-cells that recognized self-antigens *in vivo* [3, 41].

Furthermore, the inherent nature of density calculations in the plots suggests a pronounced clustering of cells at angles of 0° and 90°, with apparent sparsity of transitioning cells between these values, which is due to the nature of the density plot, rather than biological finding, as shown below.

These limitations prompted us to formally develop and establish a robust data categorization approach to Timer fluorescence data, designated as the Tocky Locus approach, as a more biologically grounded and quantitative framework for analysing Timer Angle data.

### Timer ODE modelling illuminates biological meaning of Tocky angles

To clarify the biological meaning underlying Timer Angle measurements from Tocky systems, we performed kinetic ODE simulations of Timer protein maturation under two distinct transcriptional scenarios: a constant transcription model representing sustained gene expression, and a transient transcription model mimicking a short-lived burst of transcriptional activity, as experimentally observed for Nr4a3 mRNA dynamics previously [3].

**Figure 4:**
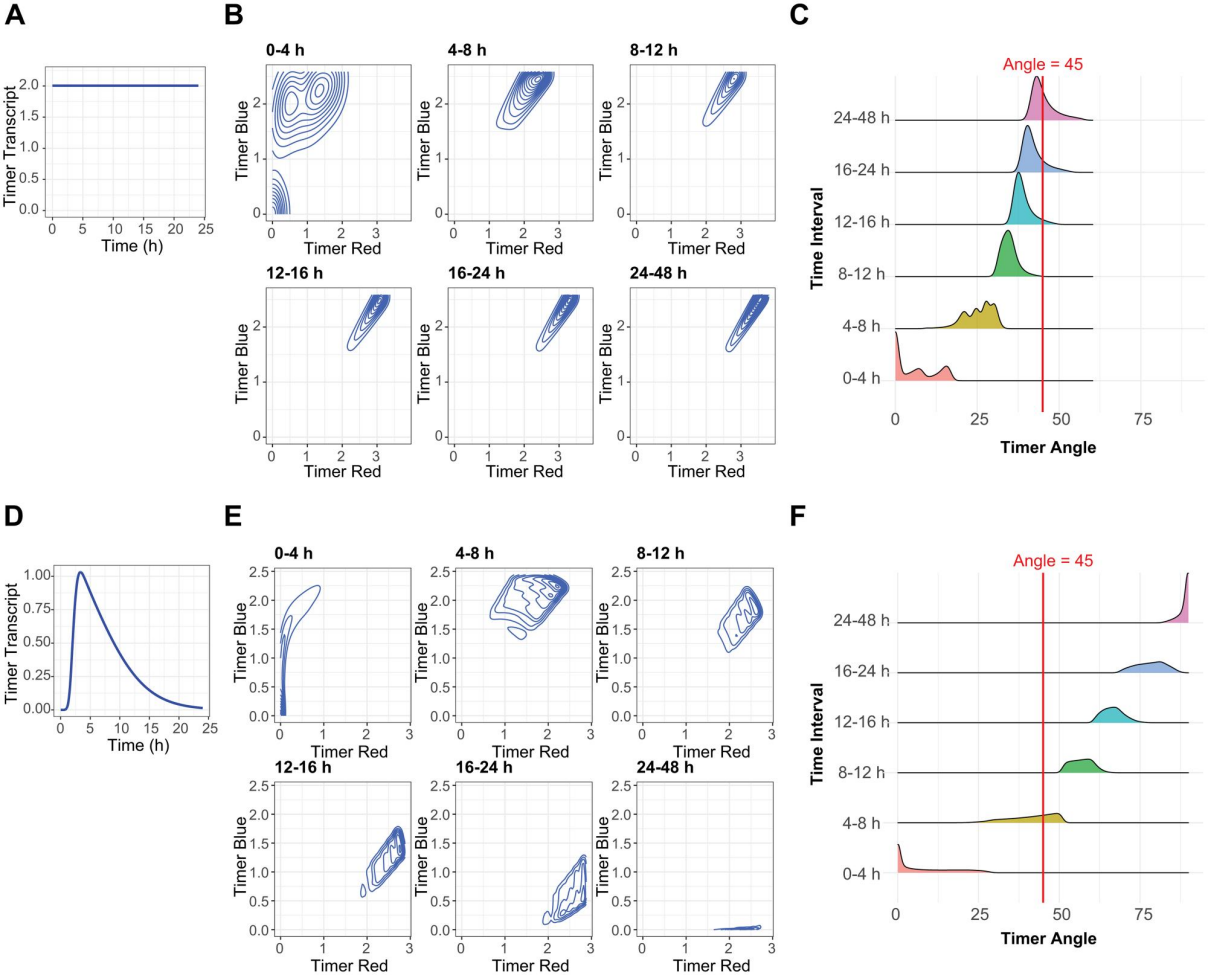
Simulation of Timer transcription and maturation dynamics. (**A**) Simulated Timer transcript levels under a constant transcription model for **B–C**. (B) Contour plots of Timer-Blue versus Timer-Red fluorescence under constant transcription across the indicated time intervals. (C) Corresponding Timer Angle distributions for the constant transcription model for **D–E**. (D) Simulated Timer transcript dynamics under a transient transcription model fitted to experimental Nr4a3 mRNA data [3]. (E) Contour plots of Timer-Blue versus Timer-Red fluorescence under the transient transcription model. (**F**) Corresponding Timer Angle distributions for the transient transcription model, illustrating broader heterogeneity and persistence of high-angle cells even at later time points

In the constant model (Fig. 4A–C), timer transcript levels remain steady over time (Fig. 4A), leading to continuous accumulation of Timer Blue and Timer Red fluorescence signals (Fig. 4B). The Timer Angle distributions under this model shift gradually from low angles (blue-dominated) towards higher angles (red-dominated), ultimately stabilizing into a unimodal distribution that reflects balanced maturation kinetics (Fig. 4C).

In contrast, the transient model (Fig. 4D–F) produces a rapid rise and subsequent decay in transcript levels, resulting in lower overall Timer protein accumulation (Fig. 4D). Consequently, Timer fluorescence progressively matures from 0° to 90° (Fig. 4E). Accordingly, cells distributed across both low and high Timer Angles, with greater variability across the time intervals (Fig. 4F). These transient dynamics generate diverse Timer Angle trajectories, reflecting heterogeneous transcriptional histories within the cell population.

### Comparison of experimental timer angle data with simulated models

**Figure 5:**
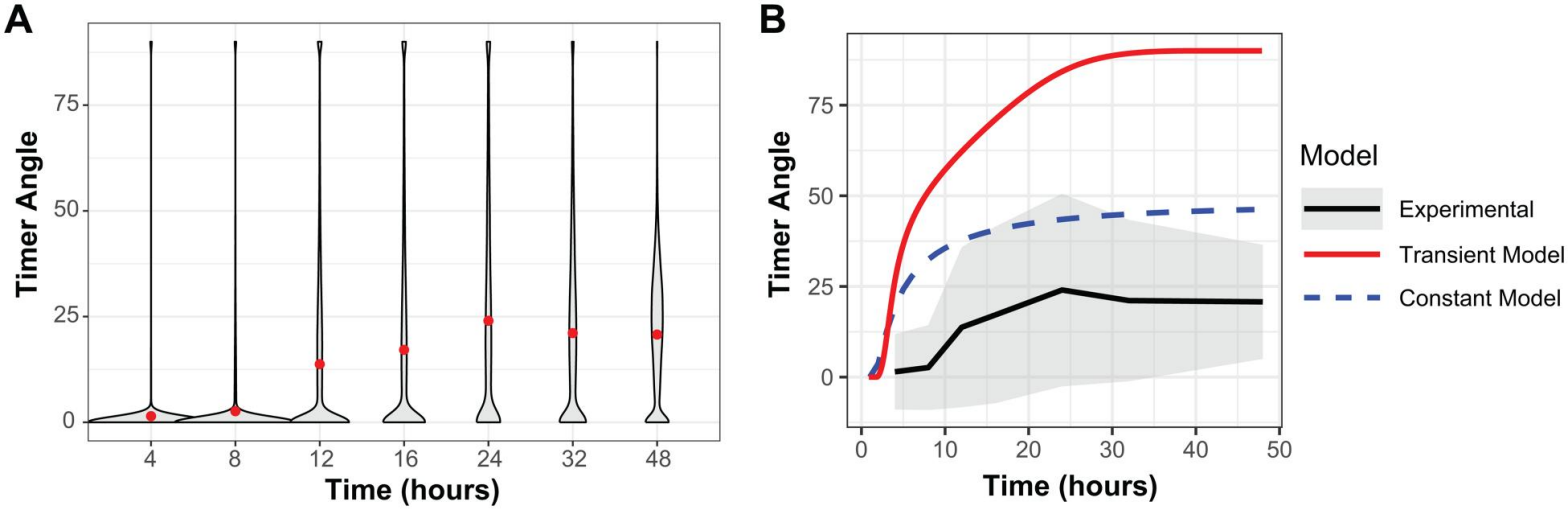
Comparison of experimental Timer Angle kinetics with ODE-simulated models. (**A**) Violin plot of experimental data showing broad distributions of Timer Angle in activated T cell samples. Mean Angle values are shown by red closed circles. (**B**) Experimental mean Timer Angle dynamics (black solid line) are shown over time, with grey shading representing the mean ± standard deviation at each time point. Simulated kinetics from the constant transcription model (dashed line) and transient transcription model (uppermost solid line) are overlaid for comparison

Notably, the variations of experimental Timer Angle in the Nr4a3 dataset are substantial, reflecting significant biological heterogeneity within cultures of activated T cells (Fig. 5A). Comparison of experimental Timer Angle data with ODE-simulated kinetics showed that the constant transcription model more closely approximates the experimental mean Timer Angle than the transient transcription model (Fig. 5B). The root mean squared error (RMSE) was lower for the constant model (23.40) than for the transient model (52.48), indicating a better quantitative fit. Nonetheless, Timer Angle values in the experimental data consistently remain lower than those predicted by either model. This difference likely reflects biological asynchrony, as not all T cells *in vitro* encounter antigen and activate transcription simultaneously [3]. Consequently, new cells continue to enter the response over time, maintaining a significant proportion of cells at low Timer Angles (Fig. 5A). Such delayed activation events are absent in both pure models, which assume synchronous transcriptional onset. Still, the modelling analysis and experimental validation together support that Timer transcription is predominantly sustained in the majority of T cells in culture.

### Tocky Locus approach to simulated models

**Figure 6:**
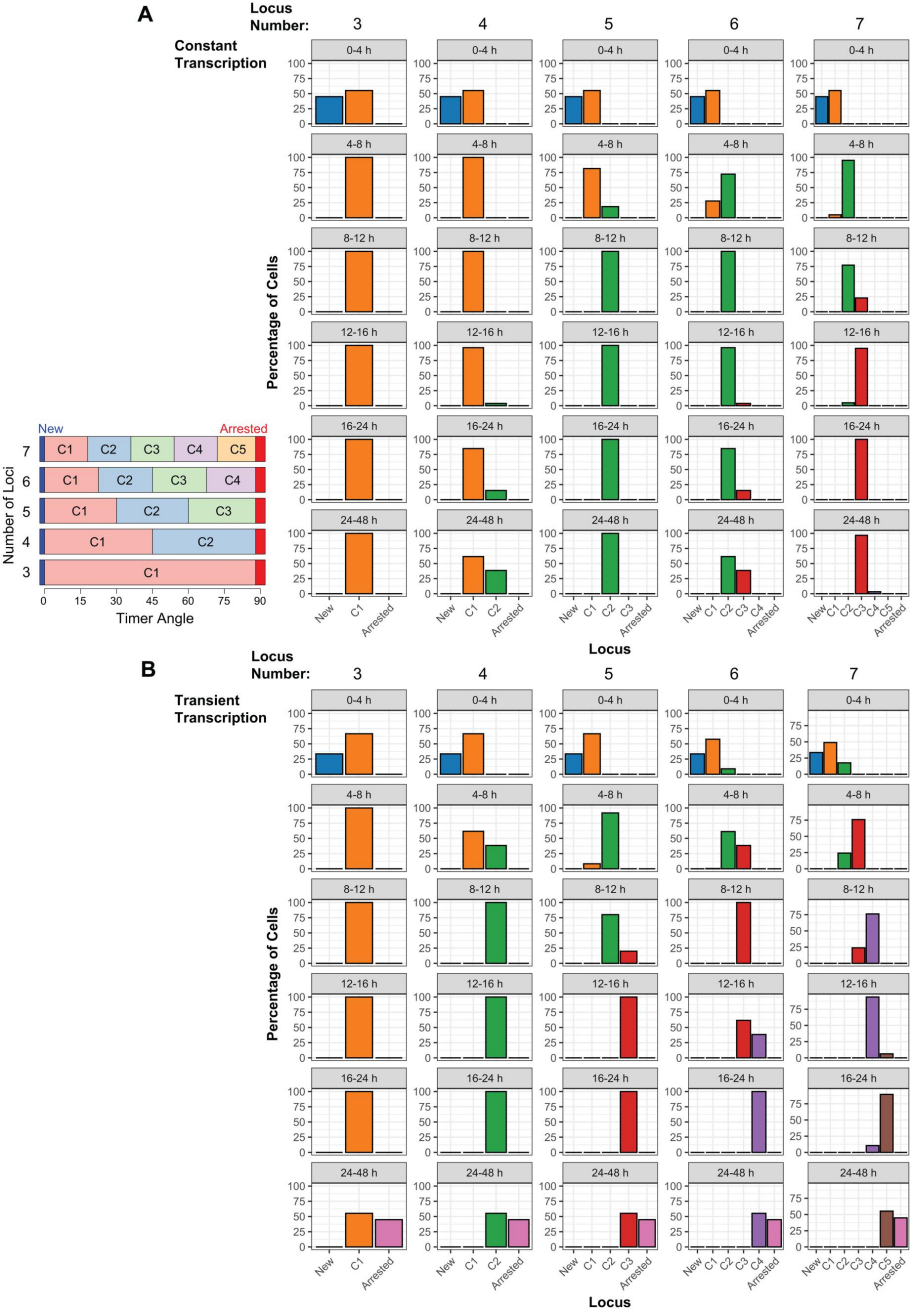
Comparison of cell distributions across Tocky loci under constant and transient transcriptional dynamics. (**A**) Simulated data in Fig. 4A (under a constant transcription model) was analysed by the Tocky Locus approach, utilizing various locus numbers. Bar plots show the percentage of cells assigned to each Tocky locus across different time intervals (rows) and binning schemes using 3–7 loci (columns). (**B**) Simulated data in Fig. 4B (under a transient transcription model) was analysed by the Tocky Locus approach, utilizing various locus numbers. Bar plots show the percentage of cells assigned to each Tocky locus across different time intervals (rows) and binning schemes using 3–7 loci (columns)

We applied the Tocky Locus categorization to simulated Timer Angle data under both constant and transient transcription models to assess how different numbers of loci capture the simulated Timer Angle dynamics (Fig. 6).

For the constant transcription model (Fig. 6A), the majority of cells accumulated in midrange loci corresponding to persistent transcription (e.g. C1 in the three-locus scheme or C2 in the five-locus scheme), consistent with sustained Timer protein expression leading to angles near 45°. Under this model, higher odd numbers of loci (i.e. 5 and 7) produced more intuitive results, showing clear accumulation of cells in the central loci (i.e. C2 and C3 in the five- and seven-locus schemes, respectively) over time.

In contrast, the transient transcription model (Fig. 6B) revealed broader shifts in locus distributions across time points. Early time intervals showed enrichment in low-angle loci (New or C1), whereas later time points exhibited substantial accumulation in high-angle loci corresponding to arrested transcription (e.g. Arrested or C5 in the seven-locus method). Thus, the locus approach successfully captured the rapid progression of Timer Angle under transient expression in a succinct manner.

Notably, increasing the number of loci improved the resolution of dynamic transitions, particularly in the transient model, where intermediate loci captured gradual shifts between New and Arrested states, providing higher granularity.

These simulations highlight the biological interpretability of Tocky Locus binning, enabling the concise capture of different transcriptional kinetics as distinct and quantifiable locus patterns that can be directly linked to underlying biological transcriptional dynamics.

Although real-world transcriptional dynamics are more nuanced and varied than either of these extreme scenarios, these models illustrate several biologically relevant principles:

1. Sustained transcription leads to accumulation of cells with Timer Angles near 45°, reflecting steady production and maturation of the Timer protein. Notably, even transiently induced cells pass through the region around 45°, but significant accumulation near 45° strongly indicates recent sustained transcriptional activity.
2. In transient scenarios, cells can traverse the entire Timer Angle range (0°–90°) within roughly 24 h. However, the progression slows at later stages, causing greater accumulation of cells near higher angles, including around 90°, compared to earlier stages.
3. Accumulation of cells near 90° indicates historical expression without recent transcriptional activation.
4. Very low Timer Angles (close to 0°) correspond to newly expressing cells. Even low but nonzero angles suggest that a cell has recently initiated transcription and begun accumulating Timer protein.

Taken together, these simulations demonstrate that the accumulation of cells with certain Timer Angle values may reveal their unique biological significance. Given the progression of Timer Angle, to categorize Timer Angle into biologically distinct phases, or loci. This provides a strong biological rationale for subdividing Timer Angle space into multiple loci (e.g. five or more), enabling discrimination between newly activated cells, cells undergoing transient dynamics, and those with sustained transcriptional histories in T cell populations. Importantly, these modelling results informed the angle boundaries used to define biologically meaningful Tocky loci, as detailed in the next section.

### A biological model of timer fluorescence data

Simulation analysis above indicated the continuous nature of Timer Angle progression and the promise of the data categorization approach to understand the dynamics of Angle values.

#### New locus

Considering that the Timer blue chromophore matures into the red chromophore with a half-life of 4 h, Timer Blue+ Red- cells indicate that these cells have recently activated Timer transcription and expressed the Timer protein.

#### Persistent locus

Continuous transcriptional activities result in an accumulation of cells towards a Timer Angle of 45°, representing the steady state of transcriptional dynamics [3].

#### Arrested locus

Timer Blue- Red+ cells are those that have once activated Timer transcription and accumulated some levels of Timer red proteins but have ceased Timer transcription long enough to allow the decay of Timer blue fluorescence.

### Optimizing the Tocky Locus categorization using hybrid timer data from real-world ata and simulated spiked-in cells

While the five-locus framework offers strong biological interpretability, the optimal number of loci for robust statistical analysis may vary. We therefore performed simulations to test different binning schemes ranging from three to seven loci.

To evaluate how the choice of locus segmentation affects the sensitivity of the Tocky Locus framework, we conducted simulation analyses using synthetic mixtures of background and spike-in cells derived from real Tocky datasets.

**Figure 7:**
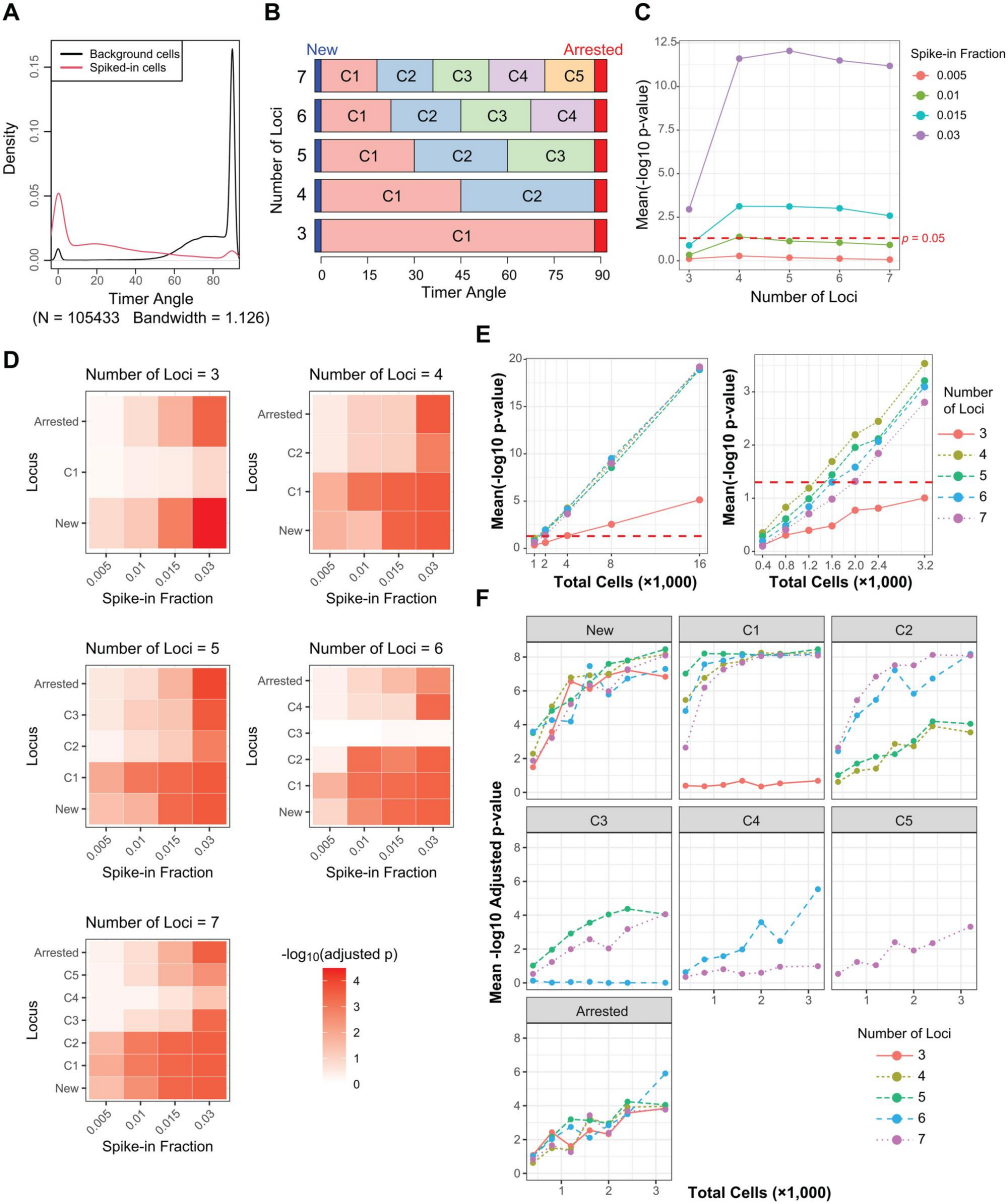
Simulation analysis of Tocky Locus performance under spike-in conditions. (**A**) Density plots of Timer Angle distributions in background and spike-in cells. (**B**) Schematic representation of data categorization into different numbers of loci. (**C**) Mean $-{\;\text{log}\;}_{10}(p)$ from chi-square tests assessing global differences in locus distributions between spike-in and background samples across varying numbers of loci and spike-in fractions. (**D**) Heatmaps of locus-wise mean $-{\;\text{log}\;}_{10}$ adjusted *P*-values across spike-in fractions for each binning scheme. (**E**) Mean $-{\;\text{log}\;}_{10}(p)$ from chi-square tests as a function of total cell number, illustrating the statistical penalty for higher binning schemes at lower cell counts. (**F**) Mean $-{\;\text{log}\;}_{10}$ adjusted Wilcoxon *P*-values assessing per-locus differences between spike-in and background samples, plotted across total cell numbers for each locus and binning scheme

In these simulations, we used relatively large numbers of cells (total cells = 10 000) and systematically varied both the number of loci used to partition Timer Angle data and the fraction of spike-in cells introduced into the background population (Fig. 7A). Specifically, background data were constructed by pooling cells from the 32-h and 48-h time points after washing and applying anti-MHC antibody to block TCR signalling (the 32.aMHC and 48.aMHC conditions), while spike-in cells were sampled from the 24-h samples shown in Fig. 3.

The number of loci was tested from three to seven, retaining the categories New (0°) and Arrested (90°), while subdividing the intermediate angle range into additional equal-width bins (Fig. 7B). This design aimed to test whether increasingly fine-grained categorizations of the Timer Angle distribution could enhance sensitivity for detecting differences.

Applying chi-square tests to the simulated datasets, the mean $-{\;\text{log}\;}_{10}(p)$ values revealed a substantial reduction in *P*-values when increasing the number of loci from three to four. Beyond four loci, further increases in segmentation had minimal impact on overall statistical sensitivity (Fig. 7C). Heatmap analysis of $-{\;\text{log}\;}_{10}$ adjusted *P*-values from locus-wise Wilcoxon analyses showed that increasing the number of loci improves performance in capturing granular changes in angle distributions (Fig. 7D).

Obviously, increasing the number of loci inevitably decreases the number of cells per locus. To examine how cell number influences detection sensitivity, as defined above, we titrated the total cell counts and observed that higher binning schemes (locus number ≥5) became increasingly penalized as total cell numbers fell below several thousand (Fig. 7E). This reflects a statistical trade-off: although finer segmentation allows detection of subtle shifts in Timer Angle distributions, it increases the number of degrees of freedom in global tests such as the chi-square test, which can inflate *P*-values if bins become sparse.

Interestingly, this penalty was not apparent in locus-wise Wilcoxon analyses (Fig. 7F), where mean $-{\;\text{log}\;}_{10}$ adjusted *P*-values for each locus remained relatively stable across different binning schemes, even at lower cell numbers. These per-locus tests are less affected by sparse bins because they evaluate local shifts in proportions rather than fitting an entire contingency table.

Moreover, although four loci appear statistically sufficient, the five-locus scheme offers biological advantages because it uniquely partitions the intermediate angle region around 45°, which is not well captured in the four-locus configuration (Fig. 7B). Consistently, the three-locus approach, which is equivalent to the common quadrant analysis of Timer Blue and Red fluorescence, is markedly underpowered for capturing dynamic changes (Fig. 7C–F). In our simulations, the five-locus scheme performed competitively in detecting changes in specific loci, particularly in regions corresponding to C1 and C3 (Fig. 7F), suggesting that this binning may better resolve subtle biological dynamics without substantial loss of statistical power under conditions of adequate cell numbers.

However, excessive increases in the number of loci can result in empty or sparsely populated bins, particularly in datasets with fewer cells or when analysing rare cell populations. Such empty bins may reduce statistical reliability and complicate interpretation, emphasizing the need for balance between resolution and practical sample size.

Overall, our results indicate that four loci are minimally sufficient for robust detection in Tocky Locus analyses. However, given the biological significance of the Persistent locus, defined around a Timer Angle of $45^{{}^{\circ}}$, the five-locus approach appears the most effective. This categorization not only enables precise identification of cells within the Persistent locus but also maintains a manageable number of categories, which is crucial for ensuring robustness in downstream statistical analyses.

### Application of Tocky Locus to two-group analysis: the OX40 dataset from Foxp3-Tocky mice

Informed by the simulations above, we examined the impact of varying locus numbers in the OX40 experimental dataset. This dataset investigated the effect of anti-OX40 antibody treatment in Foxp3-Tocky mice. In this model, mice were first sensitized on the abdominal skin with the hapten oxazolone. Three mice subsequently received anti-OX40 antibody treatment, while four mice received an isotype control antibody. Five days after sensitization, the mice were challenged on their ear skin with a small dose of oxazolone, and skin-infiltrating T cells were analysed 96 h later by flow cytometry for Fluorescent Timer profiles [9].

#### Biological hypothesis for group differences

**Figure 8:**
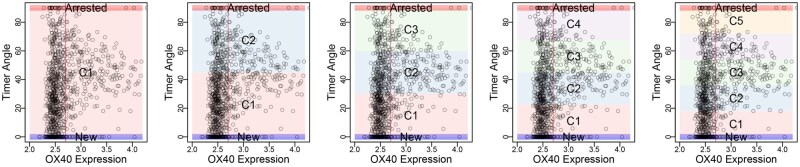
Overlay of Tocky Locus Boundaries on OX40 vs. Timer Angle Scatterplots. Scatterplots show single-cell OX40 expression versus Timer Angle in the OX40 dataset. Each panel overlays colour-coded rectangles corresponding to different Tocky Locus categorizations (ranging from 3–7 loci) to illustrate how cell distributions align with locus boundaries. The vertical lines indicate the threshold value for OX40 positivity

Biologically, it was expected that anti-OX40 treatment would specifically reduce OX40-high cells [9]. The overlay of locus categorization in OX40-Timer Angle scatterplots shows that OX40 expression is visible in cells in the Persistent (C2) and PA-t (C3) loci in the five-loci approach. Correspondingly, we expected reductions in analogous loci across different binning schemes: C1 in the three-loci scheme; C1 and C2 in the four-loci scheme; C2 and C3 in the six-loci scheme; and C2, C3, and C4 in the seven-loci scheme (Fig. 8). Accordingly, we hypothesized that anti-OX40 treatment would reduce T cells in these loci.

#### Statistical analysis of group differences

**Figure 9:**
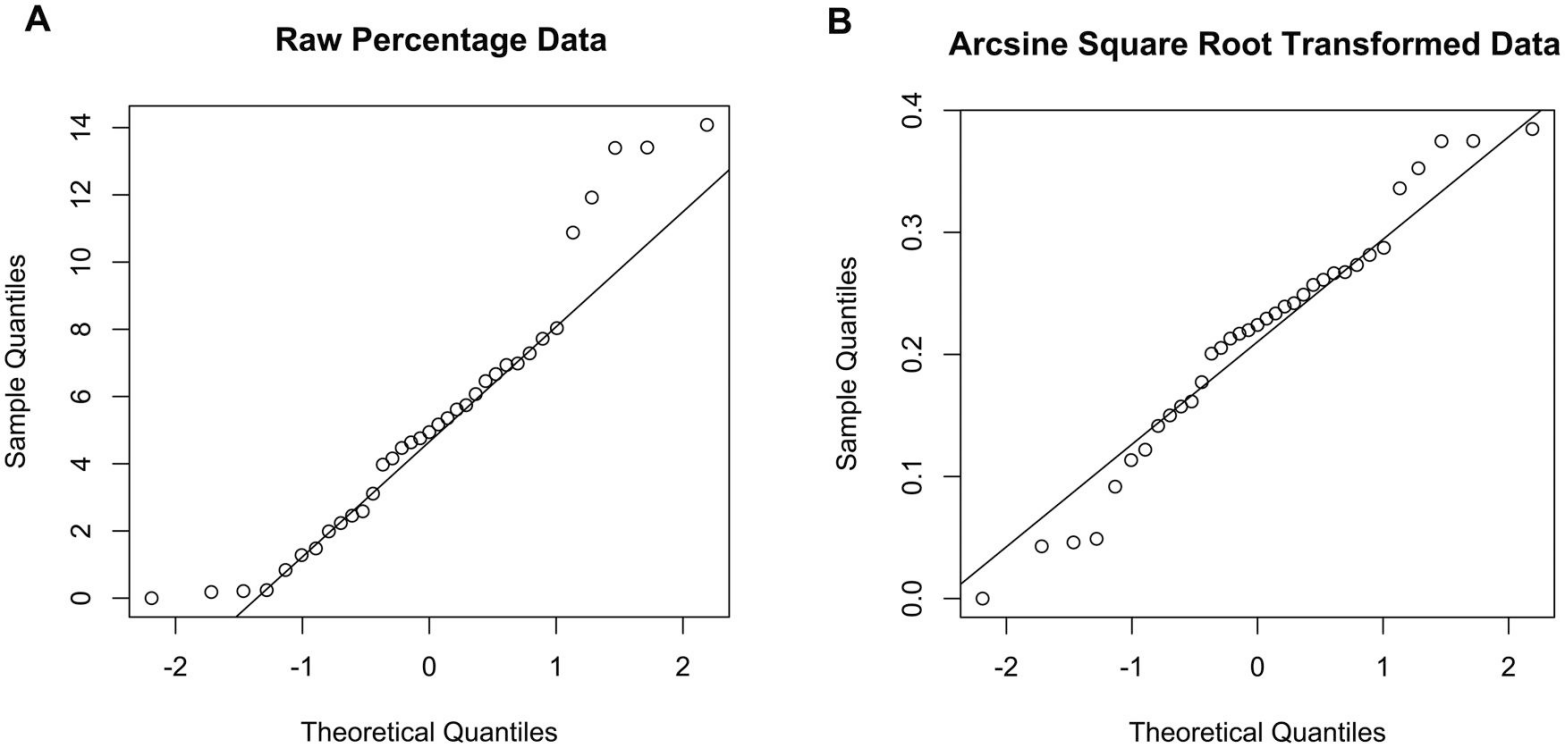
Effect of Data Transformation. (A) QQ plot of raw (untransformed) locus proportion data, showing deviation from normality. (B) QQ plot after applying the arcsine square root transformation to the same data, improving normality for statistical analysis.

To quantitatively test differences across Timer loci, we performed two levels of statistical analyses. First, we assessed differences at the locus level. Data were transformed using the arcsine square root transformation, and normality was confirmed by the Shapiro-Wilk test (Fig. 9). Subsequently, t-tests were conducted, with *P*-values adjusted using the False Discovery Rate (FDR) to account for multiple comparisons across the five loci.

**Figure 10:**
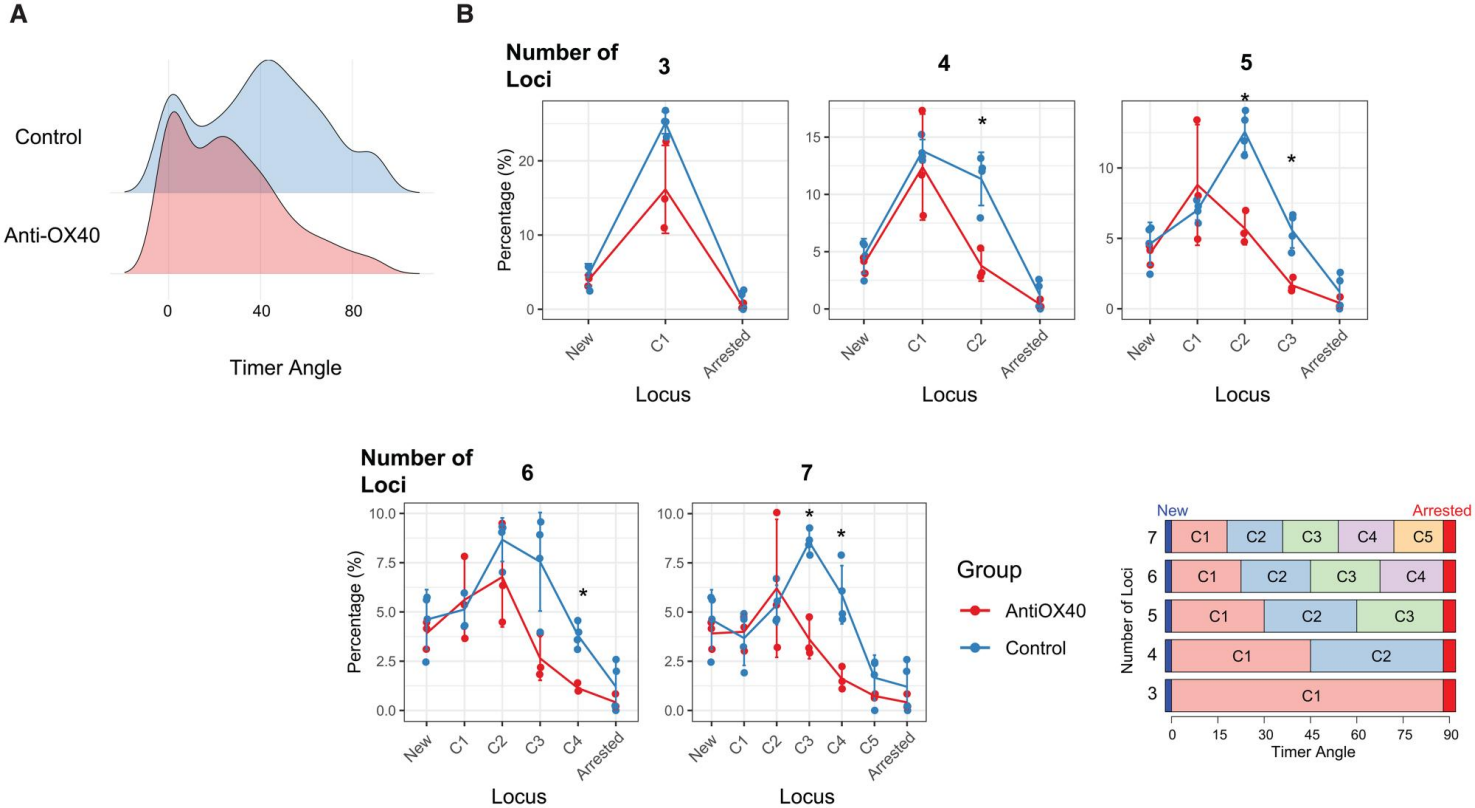
Impact of the Number of Loci on Analysis of the Anti-OX40 Dataset. (**A**) Density plot showing the distribution of Timer Angle values for anti-OX40-treated and control samples. (**B**) Tocky Locus analyses performed using 3, 4, 5, 6, or 7 loci. Data points represent the mean percentage of cells within each Tocky locus across mice in each treatment group, and error bars indicate the standard deviation among each group of mice (N=3 for Anti-OX40 and N=4 for Control). Asterisks indicate significant differences between groups with an adjusted *P*-value < .05, based on the Mann-Whitney test. The original experimental dataset was reported in [9]

Anti-OX40 induces a visible shift in the Timer Angle distribution (Fig. 10A). Accordingly, we performed a holistic comparison using a chi-square test of independence to evaluate whether the overall distribution of cells across Timer loci differed between anti-OX40-treated and control groups. This test revealed a highly significant difference in the overall distribution (X-squared =81.204,df = 4,  *P*-value < 2.2e-16).

Figure 10 summarizes analyses across varying numbers of loci. Notably, the five-locus approach captured significant shifts in cell distributions, specifically identifying reductions in the C2 (Persistent) and C3 (PA-t) categories in the anti-OX40 group. This reflects biological depletion of OX40^hi^ Foxp3+ T cells, which are highly enriched in these loci under steady-state or inflammatory conditions [9]. In contrast, analyses using three or six loci were less sensitive to these specific changes, supporting the five-locus scheme as an optimal balance between biological resolution and statistical power.

Taken together, these results highlight the utility of the Tocky Locus framework in revealing both global and locus-specific effects of immunomodulatory treatments. The significant chi-square result confirms that anti-OX40 treatment leads to an overall reshaping of Timer Angle distributions, while pairwise locus-level tests elucidate which specific transcriptional states are most affected.

### Application of Tocky Locus to two-group analysis: the CNS2 KO Foxp3-Tocky dataset

**Figure 11:**
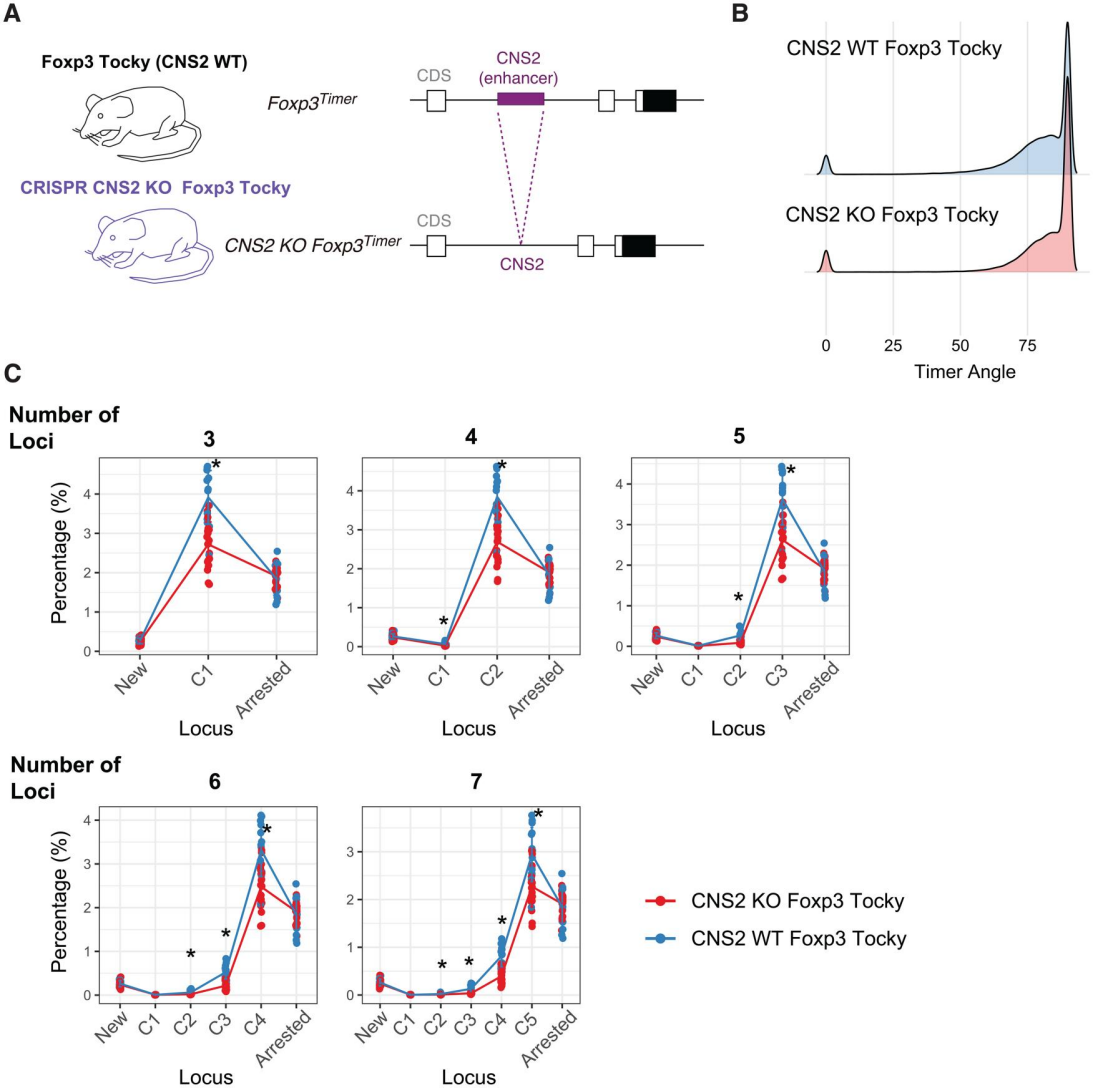
Impact of the Number of Loci on Analysis of the CNS2 KO Foxp3-Tocky Dataset. (**A**) Schematic illustrating CRISPR-mediated deletion of the CNS2 enhancer in Foxp3-Tocky mice, as previously reported [15]. (**B**) Density plots showing the distribution of Timer Angle values in CNS2 KO Foxp3-Tocky (KO) and wild-type Foxp3-Tocky (WT) samples. (**C**) Tocky Locus analyses performed using 3, 4, 5, 6, or 7 loci. Data points represent the mean percentage of cells within each Tocky locus across individual mice in each group, with error bars indicating the standard deviation among mice (N=14 for WT and N=20 for KO). Asterisks indicate significant differences between groups with an adjusted *P*-value < .05, based on the Mann-Whitney test. The original experimental dataset was reported in [15]

To further demonstrate the applicability of the Tocky Locus framework across diverse biological contexts, we analysed a new experimental dataset using Foxp3-Tocky mice with CRISPR-mediated deletion of the Conserved Non-coding Sequence 2 (CNS2) enhancer (Fig. 11). CNS2 is a critical enhancer for maintaining Foxp3 transcription. Thus, presumably the deletion of CNS2 shifts the Timer Angle distribution towards higher angles, but it is not obvious which parts of the Timer Angle data are affected.

Angle density plot showed minimal visible changes by the CNS2 deletion (Fig. 11B). We performed Tocky Locus analyses using 3 to 7 loci, and consistently observed significant differences between CNS2 KO and WT groups, particularly evident under the five-locus scheme (Fig. 11C). These results illustrate that the five-locus approach can sensitively detect subtle transcriptional dynamics even in complex biological perturbations, supporting its generalizability and robustness beyond the previously analysed OX40 and Nr4a3 datasets.

### Implementation of the five-loci Tocky Locus analysis

The analyses above support the five-locus approach, designated as the Tocky Locus approach, as a balanced method that achieves both biological relevance and robust statistical performance. Thus, we implemented the five-locus framework in the TockyLocus R package, as described below.

The Tocky Locus approach differs fundamentally from clustering methods in that it relies on biologically defined angle boundaries rather than data-derived clusters. This enables unbiased statistical comparison of continuous Timer Angle data and allows key ranges of these data to be directly associated with the underlying biological model of Tocky loci.

This section describes the key implementation features of the TockyLocus package, which incorporates this integrated biological-statistical framework.

### Data preprocessing for Tocky Locus approach and definition of Tocky Loci

**Figure 12:**
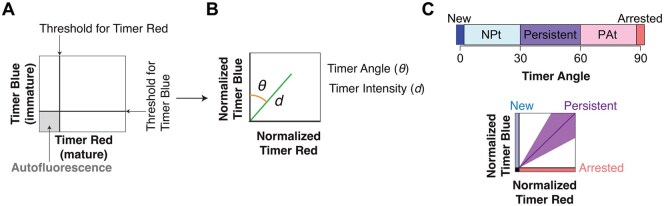
Data preprocessing and definition of Tocky loci. (**A**) Initial gating thresholds are set for Timer-Blue and Timer-Red fluorescence to exclude autofluorescence, using the prep_tocky function in TockyPrep. (**B**) After normalization of Timer-Blue and Timer-Red fluorescence intensities, Timer Angle (θ) and Timer Intensity (*d*) are calculated using trigonometric transformation. (**C**) (Upper) Timer Angle space from 0° to 90° is partitioned into five biologically defined loci representing New, New-to-Persistent transitioning (NP-t), Persistent, Persistent-to-Arrested transitioning (PA-t), and Arrested transcriptional states. These loci are visualized both as angular segments and as corresponding regions in the Timer-Blue vs. Timer-Red fluorescence space. (Lower) The conceptual Tocky Loci are schematically shown in the normalized Timer fluorescence space

Timer Angle data are categorized into five Tocky loci, each reflecting distinct biological states (Fig. 12A–C). To apply this approach, data preprocessing using the TockyPrep package is essential. TockyPrep implements several critical steps: first, thresholds for Timer Blue and Timer Red fluorescence are established to reliably exclude autofluorescence, which arises from intrinsic cellular components such as NADPH [42] (Fig. 12A).

Following thresholding, fluorescence data are normalized for both Timer Blue and Timer Red fluorescence to correct for any systematic bias between the two variables, which is essential for a robust transformation into Timer Angle values (Fig. 12B). This normalization enables calculation of two derived metrics: Timer Angle (θ), representing the angular orientation in the transformed fluorescence space, and Timer Intensity (*d*), representing the magnitude of the fluorescence vector.

Finally, the continuous Timer Angle space from 0° to 90° is subdivided into defined intervals that constitute the five Tocky loci: New, NP-t (New-to-Persistent transitioning), Persistent, PA-t (Persistent-to-Arrested transitioning), and Arrested (Fig. 12C). These loci correspond to distinct transcriptional states and facilitate biological interpretation as well as statistical comparison across experimental conditions.

- **New:** Timer Angle exactly $0^{{}^{\circ}}$. Represents newly activated cells that have just begun transcribing the Timer gene and have accumulated only unmatured (blue) Timer protein.
- **New-to-Persistent Transitioning (NPt):** Timer Angle $>0^{{}^{\circ}}$ and $\leq 30^{{}^{\circ}}$. Cells in this locus are transitioning from new expression towards steady-state transcription, indicating recent activation with ongoing Timer production.
- **Persistent:** Timer Angle $>30^{{}^{\circ}}$ and $\leq 60^{{}^{\circ}}$. Reflects cells undergoing sustained transcription, characterized by balanced maturation of Timer Blue and Timer Red proteins, typically accumulating around $45^{{}^{\circ}}$.
- **Persistent-to-Arrested Transitioning (PAt):** Timer Angle $>60^{{}^{\circ}}$ and $<90^{{}^{\circ}}$. Represents cells in which Timer transcription is declining or has ceased recently, but matured Timer Red protein is still present.
- **Arrested:** Timer Angle exactly $90^{{}^{\circ}}$. Denotes cells that have ceased Timer transcription long enough for Timer Blue protein to decay, leaving only the mature red signal.

### Visualization of Tocky locus in flow cytometric timer plots as quality control

**Figure 13:**
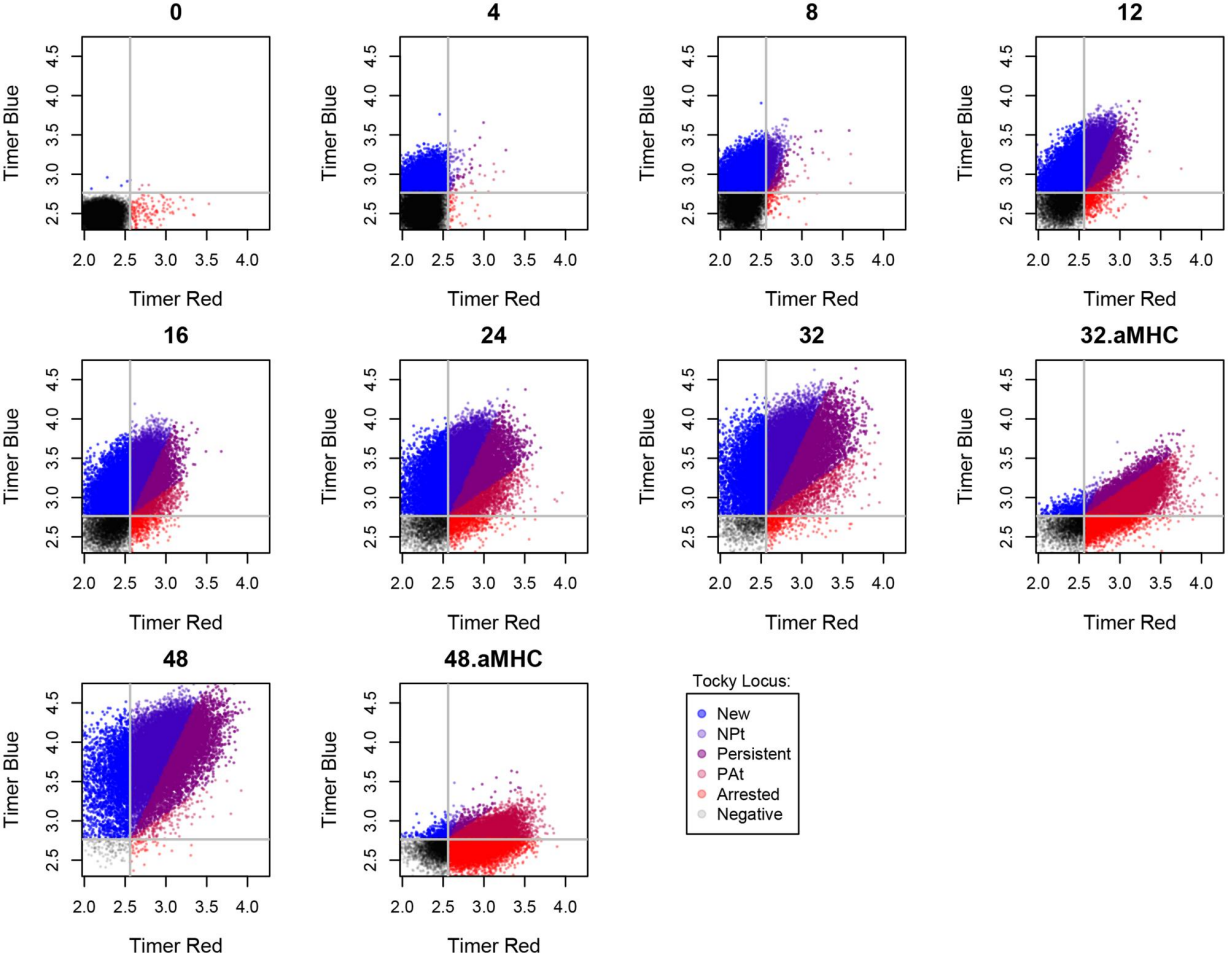
Tocky Locus Plot for QC. The Nr4a3 Tocky Dataset was analysed using the plot_tocky_locus function from the TockyLocus package. Timer fluorescence data were categorized into the five Tocky loci. The percentage of cells among CD4+ T cells in each Tocky Locus is shown, demonstrating the distribution across different loci. See Table 1 for sample definitions

For quality control purposes, the TockyLocus package facilitates the visualization of Tocky Locus categorization in flow cytometric plots displaying Timer blue and red fluorescence (Fig. 13). These plots successfully identify Timer autofluorescence (shown in grey) and the five Tocky loci, which are demonstrated using distinct colours in Fig. 13.

### Quantification of timer angle dynamics by Tocky Locus plot

**Figure 14:**
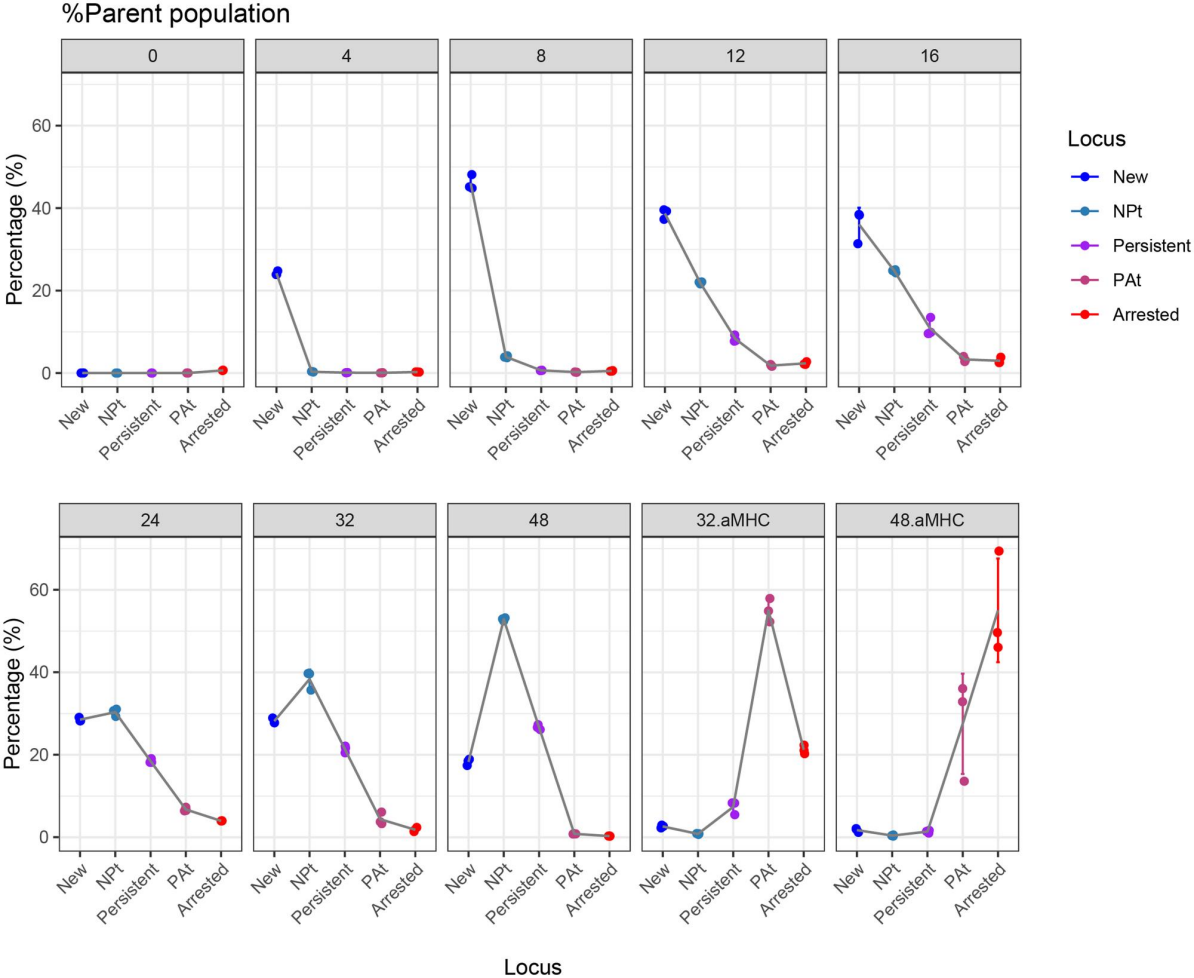
Tocky Locus Plot: This plot visualizes the dynamic progression of Tocky Loci post-antigen stimulation, illustrating how T-cells transition through various states of Timer protein expression over time. The percentage of cells among CD4+ T cells in each Tocky Locus is shown, demonstrating the distribution across different loci. See Table 1 for sample definitions. Data points show mean percentages across three cell cultures, and error bars represent standard deviation (N=3)

For the quantitative analysis of Timer fluorescence dynamics, the quantification of cells within each range of Timer Angle proves to be effective. As demonstrated in Fig. 14, the PlotTockyLocus function facilitates both the quantification and visualization of Tocky Loci in a streamlined manner. The five Tocky loci successfully capture the dynamics of Timer fluorescence, revealing that T-cells express the Timer protein as early as 4 h post-antigen stimulation. Over time, Timer fluorescence matures and more cells are categorized into advanced Tocky Loci, including NP-t, Persistent, and PA-t. Following the termination of stimulation, there is a rapid increase in Timer Angle, transitioning the majority of cells to PA-t and subsequently to Arrested within 8 and 24 h, respectively.

## Discussion

The primary challenge in using Fluorescent Timer data lies in its analysis. Most current studies rely on MFI-based methods [34–40]. Alternatively, traditional approaches use arbitrary gating or quadrant-based classification, segmenting fluorescence into Blue${\;}^{+}$Red${\;}^{-}$, Blue${\;}^{+}$Red${\;}^{+}$, Blue${\;}^{-}$Red${\;}^{+}$, and Timer${\;}^{-}$ cells [5, 39, 40]. However, our analysis shows that these methods lack the resolution required for effective visualization and statistical interpretation.

The limitations of MFI-based analysis have indeed led to misinterpretations in published studies. For example, Elliot et al. (2021) [39] examined checkpoint blockade effects using the Nr4a3-Tocky system, gating exclusively on Timer Blue-positive cells and comparing Timer Blue MFI between groups (see Fig. 5 in the publication). This led the authors to interpret increased Blue MFI under treatment as evidence of heightened T cell reactivation, effectively reducing the potential power of the Tocky system to a singular, qualitative metric. However, this approach failed to capture the effects of treatments on the full dynamics of Timer fluorescence, particularly neglecting changes in transcriptional duration, which cannot be adequately assessed using single Timer Blue MFI values alone.

In contrast, the Tocky Locus framework analyses the entire cell population by partitioning continuous Timer Angle values into biologically defined loci, enabling a more precise and unbiased assessment of transcriptional dynamics with quantitative and statistical analysis methods.

The TockyLocus approach circumvents these limitations by applying a biologically informed categorization scheme to discretize Timer Angle into five angular intervals. This allows standardized quantification of dynamic cellular states while avoiding arbitrary thresholds or assumptions of distribution modality. Our implementation builds on prior work in trigonometric transformation of Timer data [3], extending it to provide a reproducible framework for discretization, visualization, and statistical analysis. Unlike manual gating, the Tocky Locus system identifies edge-state populations (New, Arrested) as fixed anchors and segments the intermediate Timer-positive states in a reproducible and interpretable fashion. Doing so, the Tocky Locus approach differs fundamentally from clustering methods in that it relies on biologically defined angle boundaries rather than data-derived clusters.

Unlike clustering methods, the Tocky Locus approach avoids introducing the randomness inherent in clustering algorithms, thereby ensuring that Timer Angle data analysis remains deterministic and reproducible. This enables unbiased statistical comparison of continuous Timer Angle data and allows key ranges of these data to be directly associated with the underlying biological model of Tocky loci.

Critically, we evaluated classification schemes using 3 to 7 loci and found that the five-locus model offers an optimal trade-off between resolution and robustness. Categories fewer than five failed to resolve intermediate transcriptional states (e.g. transitioning or persistent expression), while excessive segmentation introduced noise and reduced interpretability, especially in low-cell-count populations. Although our approach relies on biological rationale (e.g. 45${\;}^{{}^{\circ}}$ Timer Angle indicating sustained transcription), the discretization boundaries are analytically defined and can be generalized to other FT-based systems. This positions TockyLocus as a broadly applicable method for quantifying temporal transcriptional dynamics in FT-reporter experiments.

The five-locus approach effectively highlights Nr4a3-Tocky’s ability to capture rapid TCR signalling shut-down after antigen removal. Anti-OX40 selectively depletes Persistent/PA-t Foxp3${\;}^{+}$ Treg subsets, supporting the uniqueness of persistent Foxp3 expressors [9]. Furthermore, the current study also demonstrated that categorizing into too many loci can lead to issues with small sample sizes in each category. Thus, the current study formally confirms the validity of the Tocky Locus approach and provides robust evidence supporting this data categorization method for interpreting flow cytometric Timer fluorescence data, consistent with previous applications [3, 9, 7, 14].

The current implementation incorporates statistical methods specifically tailored to analysing percentage data within each Tocky Locus. When using the five-locus approach, it is advisable to apply *P*-value adjustment methods to address multiple testing effectively. However, analysing percentage data, particularly with small sample sizes, poses significant challenges. In response, we have expanded our statistical toolkit to include not only the non-parametric Mann–Whitney test with *P*-value adjustment but also data transformation techniques such as the arcsine square root and logit transformations. These transformations are prerequisites for applying the parametric Student’s *t*-test. The selection of a transformation method depends on the specific case and data structure. These methods are designed to enhance the precision and effectiveness of the Tocky Locus approach.

## Conclusion

In summary, TockyLocus complements existing tools for flow cytometric Fluorescent Timer analysis by offering a validated, reproducible, and biologically grounded method for analysing Timer Angle data. It improves interpretability, supports robust statistics, and eliminates the subjectivity associated with traditional gating. Together with TockyPrep, it provides a unified computational workflow to advance studies of dynamic gene expression in immunology and other time-resolved biological systems.

## Data Availability

The TockyLocus package is freely available at our GitHub site: https://github.com/MonoTockyLab/TockyLocus. Documentations can be accessed at https://MonoTockyLab.github.io/TockyLocus.
